# Diagnosing oral and maxillofacial diseases using deep learning

**DOI:** 10.1038/s41598-024-52929-0

**Published:** 2024-01-30

**Authors:** Junegyu Kang, Van Nhat Thang Le, Dae-Woo Lee, Sungchan Kim

**Affiliations:** 1 HYPERCloud, Seoul, 06038 Korea; 2 Faculty of Odonto-Stomatology, Hue University of Medicine and Pharmacy, Hue University, Hue, 49120 Vietnam; 3 The Department of Pediatric Dentistry, Jeonbuk National University, Jeonju, 54896 Korea; 4 Biomedical Research Institute of Jeonbuk National University Hospital, Jeonbuk National University, Jeonju, 54896 Korea; 5 Research Institute of Clinical Medicine of Jeonbuk National University, Jeonju, 54896 Korea; 6 The Department of Computer Science and Artificial Intelligence, Jeonbuk National University, Jeonju, 54896 Korea; 7 Center for Advanced Image Information Technology, Jeonbuk National University, Jeonju, 54896 Korea

**Keywords:** Dentistry, Diagnosis, Oral diseases, Radiography

## Abstract

The classification and localization of odontogenic lesions from panoramic radiographs is a challenging task due to the positional biases and class imbalances of the lesions. To address these challenges, a novel neural network, *DOLNet*, is proposed that uses mutually influencing hierarchical attention across different image scales to jointly learn the *global* representation of the entire jaw and the *local* discrepancy between normal tissue and lesions. The proposed approach uses local attention to learn representations within a patch. From the patch-level representations, we generate inter-patch, i.e., global, attention maps to represent the positional prior of lesions in the whole image. Global attention enables the reciprocal calibration of path-level representations by considering non-local information from other patches, thereby improving the generation of whole-image-level representation. To address class imbalances, we propose an effective data augmentation technique that involves merging lesion crops with normal images, thereby synthesizing new abnormal cases for effective model training. Our approach outperforms recent studies, enhancing the classification performance by up to 42.4% and 44.2% in recall and F1 scores, respectively, and ensuring robust lesion localization with respect to lesion size variations and positional biases. Our approach further outperforms human expert clinicians in classification by 10.7 % and 10.8 % in recall and F1 score, respectively.

## Introduction

More recently, deep neural network application has been extended to dental radiographs for oral disease diagnosis and treatment planning^1^, including tooth segmentation^2^, cervical vertebral maturation estimation^3^, landmark detection^4^, periodontal bone loss identification^5^, and jaw tumour and cyst detection^6^.

Among these, applications providing detection of radiolucent lesions for early diagnosis of jaw tumors and cysts are gaining attraction due to their significant impact on oral health^7^. During regular dental examinations, panoramic radiographs can provide meaningful information about maxillofacial structures and teeth with relatively low radiation exposure. *Ameloblastoma* (AB), *odontogenic keratocysts* (OKC) and *dentigerous cysts* (DC) are representative maxillofacial diseases that usually progress without pain or symptoms, making early diagnosis crucial^8^.

Panoramic dental radiographs have inherent limitations in that 3D objects are represented as 2D images because the facial skeleton and bony structures of the tissues are superimposed and distorted during the scan around the patient’s head. Correctly identifying jaw tumors and cysts from panoramic radiographs can be challenging even for experienced clinicians^6^. Although, for this purpose, recent methods have proposed simple modifications of convolutional neural networks (ConvNets) that are designed for object recognition tasks on natural images^6,9–12^, these methods have not adequately addressed the fact that dental radiographs are inherently different from natural images. First, cystic and tumorous jaw lesions, such as odontogenic keratocysts, are typically unilocular or multilocular cystic lesions, most commonly found in the posterior body and ramus of the mandible^13^. This results in a significant positional prior that should be exploited when training a model. Secondly, there are also non-trivial class imbalances between the lesion categories due to the different incidences of the diseases^14^. For example, AB or OKC cases are less frequent than DC cases^8^. As a result, the number of samples from AB or OKC cases in our dataset is only half that of DC cases. Finally, the rarity of the diagnosed diseases limits the acquisition of images. Worse still, the panoramic images typically have tens of millions of pixels, making it difficult to properly design neural network models. The high resolution of the images requires the model to have large receptive fields, which increases the complexity of the model and thus the need for more training data. Although the inputs can be downsampled to reduce the number of model parameters, local information about tissue texture is likely to be lost.

We address the above difficulties by proposing a novel neural network, called *DOLNet*, for the diagnosis of odontogenic lesions from a given panoramic radiograph. DOLNet consists of two stages; the first stage extracts local features from the patches of the input images. In the second stage, we aggregate the patch-level features into the representation of the whole image to learn the relationships between the patches. We aim to suppress features that are locally but not globally meaningful, and to highlight suspicious regions in the context of the learned prior knowledge of lesion locations.

In particular, the first stage of DOLNet uses *patch-level attention* to extract position-independent local features of individual patches. In the second stage, we build a *global attention map* representing the relationships between patches across the whole image. We then refine the features of the patches using the global attention maps. As a result, this *mutually influencing attention* allows the model to jointly learn the positional prior globally and the tissue representations locally.

To address the class imbalances, we propose a simple yet effective data augmentation method to increase the training samples of minor lesion categories. Our method synthesizes natural-looking abnormal cases from crops along with irregular lesion boundaries, unlike existing augmentation techniques.

Experiments indicate that our work, DOLNet, improves diagnostic performance significantly compared to previous work. When classifying three lesion types and normal cases, DOLNet outperforms the state-of-the-art approaches by a wide margin of 42.4 % and 44.2 % in recall and F1 score, respectively. Our approach also achieved an improvement in lesion localization performance of 29.7 % in intersection over unit (IoU). Moreover, we also demonstrate that the performance of our approach in lesion classification is superior to that of professional clinicians, with an improvement of 19.2 % in recall and 21.0 % in F1 score.

In summary, our contributions are as follows.

- *Mutually influencing hierarchical attention* We introduce a hierarchical attention mechanism for learning features jointly across different image scales.
- *Data augmentation for class imbalance* We create natural-looking images through the use of lesion crops of arbitrary shapes, which effectively augment the training samples of the smaller classes.
- *Diagnosis performance and practicability* Our approach achieves state-of-the-art performance, outperforming the latest methods and even expert human clinicians.

## Related work

In the following, we provide an overview of neural network-based approaches to dental image diagnosis and discuss feature learning techniques relevant to the proposed method.

### Machine learning approaches to diagnosing oral and maxillofacial diseases

Various modalities such as radiography, cone-beam computed tomography (CBCT) and magnetic resonance imaging (MRI) are used to identify lesions from dental radiographs^15^. CBCT and MRI provide 3D information for the diagnosis of odontogenic tumors that are otherwise difficult to identify^16^. Although dental radiography has poor diagnostic performance compared to CBCT^17^, it has the advantage of low radiation exposure for early diagnosis at low cost.

The classification and localization of odontogenic lesions are relatively new areas of research. To the best of our knowledge, since its first attempt^9^, subsequent approaches have followed^6,10–12,18,19^. GoogLeNet^20^ along with an additional simple branch was used to predict five lesion types and their locations as bounding boxes^10^. Recent studies have used similar models for object detection tasks. Fine-tuned YOLOv2^21^ is used to improve localization when classifying DC, OKC, AB, and normal cases^6^. Another method^11^ is built on top of YOLOv3^22^ to classify and localize four types of lesions using a feature pyramid network (FPN)^23^ and skip connections.

Although object detection is a task closely related to the problem addressed in this study, detection models such as mask R-CNN^24^ and YOLO models (YOLOv2^21^, YOLOv3^22^, and YOLOv4^25^) are not directly applicable to dental tumor diagnosis for the following reasons. First, they take whole images as input, but cannot effectively handle high-resolution images compared to patch-based approaches such as our method. Specifically, when diagnosing high-resolution medical images, the receptive field size at the last convolution layers of these models is not large enough to examine the input image as a whole, which may lead to suboptimal predictions. On the other hand, our approach takes patches as input, which allows us to adjust the patch size according to the receptive field size of the convolutional layers in our model. However, this requires additional steps to learn the relationships between patches for diagnosis considering the whole input images, which is done by the attention proposed in our approach. Second, the mask R-CNN^24^ approach used a novel loss, known as focal loss^26^, to address the class imbalance in terms of size difference between objects and background. However, object detection models typically assume a uniform distribution of object locations without accounting for spatial biases, such as those encountered in dental tumor detection.

Transfer learning has been used for lesion classification from multimodal images based on CBCT and panoramic radiographs^18^. An ensemble of two classification networks is used to identify four lesion types using a dataset of manually extracted regions of interest^12^. Hu et al. proposed a method based on self-supervised learning, which is the most relevant to ours in that it uses separate branches for classification and localization and augmented lesion patches in normal images to synthesize data samples^19^. However, this method requires a large dataset to perform self-supervised learning.

HierarchicalDet^27^, a diffusion-based model, is one of the latest approaches to dental tumor diagnosis. Although promising, this method is derived from DiffusionDet^28^, a diffusion-based object detection model that requires a large dataset. For example, the dataset used in HierarchicalDet consists of 2300 labeled images and 1571 unlabeled images, where the number of labeled images is already 4 times larger than our dataset.

### Feature learning for medical images

We present three recent advances in the learning of medical image features that are relevant to the proposed method: patch-based analysis, attention, and confusion-based data augmentation.

#### Patch-based analysis

Patch-based analysis is widely used in medical domains due to the high resolution of images in these domains. An early approach performs expectation maximization to select discriminative patches for whole-slide image (WSI) classification^29^. A WSI analysis in pathology is a typical application of a patch-based method formulated as multiple-instance learning (MIL)^30,31^, with an emphasis on various MIL pooling operations, such as attention^32,33^. They are similar to ours in learning multi-scale features from patches^34,35^, which is advantageous for learning domain-specific anatomical knowledge, e.g., cellular features to tissue phenotypes^35^. However, obtaining a sufficient number of patches containing lesions for model training is challenging in dental radiographs compared to WSIs. This is because dental lesions are typically concentrated in specific regions, and thus only a limited number of useful patches can be sampled.

Previous work assumes sufficient training data and no class imbalance or positional bias. As a result, simple merging of patch-level results is often insufficient; in this context, the mutually-influencing hierarchical attention and data augmentation are our proposed solutions to the aforementioned problems.

#### Attention

An attention mechanism allows a neural network to flexibly use the most relevant parts of the input. Attention uses a weighted combination of all encoded input vectors to assign the highest weights to the most relevant vectors. Different attention mechanisms have been proposed for learning the anatomical structures of targets of different shapes and sizes^36–38^. This is called global attention; it learns the relationship of one element in the input to that in another part. A gated attention network is such an example for learning salient features for classification of 2D ultrasound images and organ segmentation of 3D CT scans^36^ and breast cancer from histopathological images^37^. In contrast, a recent approach^38^ applied a local attention mechanism to consider the regional context of features, i.e., by using a feature pyramid network (FPN) to segment the cardiac structures from 2D echocardiography data.

A saliency map is a popular way to explain the model predictions using the pixel-level importance of an input image^39,40^. While a saliency map provides regional cues for interpreting model predictions, the direct application of existing saliency map approaches to lesion localization is limited because a saliency map does not necessarily correspond to lesions.

Transformer becomes popular for learning how elements of an input attend to each other^41–43^. Since its success in natural language processing^41^, it has been adopted in many vision tasks and has achieved state-of-the-art performance^42,43^. Swin Transformer^44^ is relevant to our work in that it can learn self-attention between pixels in patches of different scales by moving windows over an input image. While these approaches are promising, the large capacity of models based on Transformer can require a large dataset for training, which is costly and often impractical for the problems we solve.

**Figure 1:**
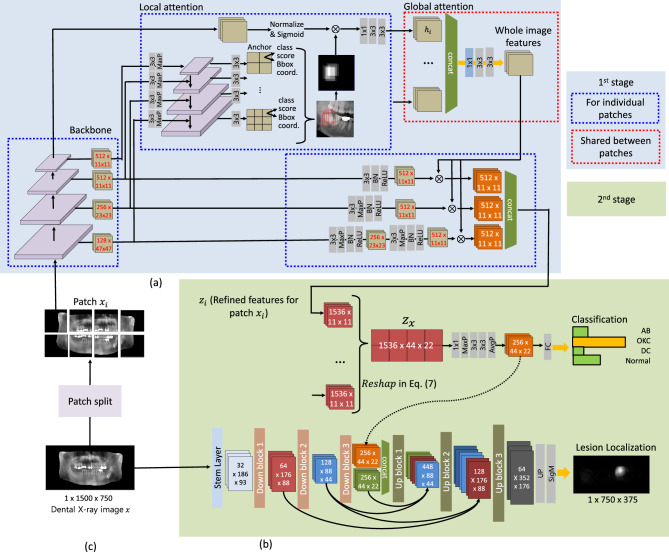
Overview of DOLNet: (**a**) the first stage for extracting patch-level representations from the proposed mutually influencing hierarchical attention. The initial patch encoding is obtained from individual local attention, and they are aggregated to create a global attention map, shown as a dotted red box, which inversely calibrates the patch representations. (**b**) The second stage corresponding to Eq. (1) with two branches for lesion classification and localization. (**c**) Preprocessed input image and patch split (best viewed in color).

#### Data augmentation based on mixup

Mixup performs a linear interpolation of two randomly selected images to train a model with additional data samples^45^. Mixup improves the robustness of a model to corrupted labels, avoids overfitting, and increases generalization. There are several variants, such as CutMix^46^ and AugMix^47^. Mixup is widely used in medical applications, such as MRI brain image segmentation^48^, as an effective sampling strategy to resolve class imbalance at different levels, including class and instance level^49^, image and object level^50^, and layer-wise feature level in latent space^51^. Inspired by mixup, we introduce a simple yet effective way to augment lesion crops in normal images to synthesize additional abnormal samples, thereby mitigating the class imbalance. Unlike previous work, our augmentation method considers arbitrary lesion shapes and thus synthesizes more natural-looking examples.

## Method

For a given dental radiograph, the goal of this paper is (1) to classify lesions contained in the image as one of three representative types “AB”, “OKC”, and “DC”, if any, and their locations in the image as bounding boxes. Otherwise, the image is classified as “Normal”. All methods were performed in accordance with the relevant guidelines and regulations.

### Model overview

For classification and localization of lesions from a dental panoramic image $x \in {{\,\mathrm{\mathbbm {R}}\,}}^{w \times h}$, DOLNet uses a neural network $f_\theta$ as

1 $$\begin{aligned} \{\hat{t}_x, \hat{s}_x\} = f_\theta ( x ), \end{aligned}$$

where $\hat{t}_x \in \{$“AB”, “OKC”, “DC”, “Normal”$\}$ is the predicted target class denoting the three odontogenic lesions and normal, and $\hat{s}_x \subset [0,1]^{w^\prime \times h^\prime }$ is a segmentation map for lesion localization. *w* ($w^\prime$) and *h* ($h^\prime$) are the width and height of *x* ($s_x$). $\theta$ is a set of trainable weights.

Figure 1 shows the overall structure of DOLNet with two stages: (1) the extraction of the patch-level representations from *K* non-overlapping patches $\{x_i\}_{i=1}^K$ of *x* with the proposed mutually influencing hierarchical attention, and (2) the classification and localization of lesions using the global heatmap learned in the first stage for the whole image. The first stage relies on three networks: a ConvNet backbone to encode $x_i$, an FPN to learn region proposals as candidate lesion locations over $x_i$, and the hierarchical attention to enhance the features of $\{x_i\}$. The patch-level analysis is performed for each of the patches independently using a single network. In other words, all patch analyses share the same parameters.

The second stage then aggregates the features of *K* patches into the global representation for *x* and performs the classification. Another branch of the network in this stage corresponds to localization, which is an autoencoder for predicting the lesion segmentation for *x*. This branch uses the global features learned by the classification branch to achieve a better segmentation. In the following section, we present the details of each stage.

### First stage: patch-based analysis

#### Backbone network

We use DenseNet-121^52^ as the backbone for encoding patches, due to its recognition performance and smaller model capacity than its competitors for a dataset used in this study. We partition the inputs into patches considering the receptive field of the backbone (447 $\times$ 447), which is much smaller than the input resolution. In other words, we set the patch size to ensure that the receptive field in the patch-level analysis network can cover the entire region of the input patch. If a model with greater capacity is used, the number of patches *K* could be reduced by increasing the resolution of the patches, i.e., larger patches. Conversely, such a design choice will require a correspondingly larger data set.

#### Local attention

Figure 1a shows the region proposal network of DOLNet for learning the patch-level representation based on local attention. We use an FPN-based regional proposal for lesion localization within a patch $x_i$. The FPN takes layer-wise features of different resolutions from the backbone to predict the bounding boxes of lesions at different scales. Since lesions can vary drastically in size and shape, multi-scale bounding box predictions in the FPN benefit robust lesion identification. In particular, we use intermediate features of three different resolutions, i.e., 11 $\times$ 11, 23 $\times$ 23, and 47 $\times$ 47, derived from the four dense blocks of DenseNet. Features with smaller resolutions can better detect larger lesions and vice versa. For each element in the features, denoted as “anchors” in Fig. 1a, the FPN predicts the confidence scores for each lesion type and their bounding box coordinates of predefined ratios, yielding many lesion proposals. We then select the top 400 proposals in the score and merge their bounding boxes to create a distribution of lesion locations as a heatmap. We denote the heatmap by $\psi ^{fpn}_i \in [0,1]^{0.25 w/K \times 0.5 h/K}$ considering that the aspect ratio between the width and height of the input patch $x_i$ is 2:1.

The heatmap represents attentive information about the lesion location in the patch and thus is therefore used to refine the features from the last layer of the backbone. This is followed by a 1 $\times$ 1 convolution and two 3 $\times$ 3 convolutions follow to reduce the feature channel size. We denote the initial feature of the patch as $h_i$,

2 $$\begin{aligned} \begin{aligned} h_i&= \left( C_3 \circ C_3 \circ C_1 \right) \left( \texttt {Sigm} \left( \texttt {Nrm} \left( B_{4} (x_i) \right) \right) \otimes \psi ^{fpn}_i \right) , \end{aligned} \end{aligned}$$

where $C_k$ is a k $\times$ k convolution, $\circ$ a function composition, Sigm a sigmoid function, Nrm a normalization, $B_l$ features from the *l*-th dense block (out of four) in the backbone, with the blocks having smaller subscripts closer to the input, and $\otimes$ the element-wise product of vectors.

#### Global attention

While the patch-level encoding in Eq. (2) may identify suspicious regions from a patch-level point of view, the global view over a whole image may lead to different (and better) predictions. Therefore, our approach aggregates the patch representations, aiming to construct the global information corresponding to the whole image, as shown in Fig. 1b. First, we concatenate the patch features $\{h_i\}$ to create a global attention map $h \in {{\,\mathrm{\mathbbm {R}}\,}}^{512 \times 11 \times 11}$ as

3 $$\begin{aligned} h = ( C_3 \circ C_3 \circ C_1 ) \left( \texttt {Concat} \left( h_1; \ldots ; h_K \right) \right) , \end{aligned}$$

where Concat is the feature concatenation. In the above equation, applying the convolutions to the concatenated features of different patches allows the model to learn non-local features at the image level.

Then, the global attention map, *h*, is used when merging the intermediate features of the backbone, $B_l(x_i)$, incorporating the different features of the different backbone layers into a common latent space as

4 $$\begin{aligned} z_i &= \texttt {Concat} (z_{i,1}; \ldots ; z_{i,K}) \otimes h, \\ z_{i,1} &= ( \texttt {ReLU} \circ \texttt {BN} \circ C_3) ( B_1 (x_i) ), \end{aligned}$$

where $z_{i} \in {{\,\mathrm{\mathbbm {R}}\,}}^{1536 \times 11 \times 11}$ is the attended feature as a result of combining $z_{i,l} \in {{\,\mathrm{\mathbbm {R}}\,}}^{512 \times 11 \times 11}$, which is transformed $B_l(x_i)$, and the global attention map *h*. ReLU is the rectified linear unit, and BN is a batch normalization. For simplicity, Eq. (4) represents only $z_{i,1}$ (see Fig. 1a for a full description). We share the global attention map *h* across all patches. We feed ${z_i}$ into the classification branch.

### Second stage: classification and localization

#### Classification

The lesion classification and localization branches of DOLNet are illustrated in Fig. 1b. The classification network is given as

5 $$\begin{aligned} t_x&= \underset{ t }{ \arg \min }~c_{t} (x), \end{aligned}$$

6 $$\begin{aligned} c_{t} (x)&= \texttt {Sm}_{t}( \texttt {Mlp}( \texttt {Avg} ( C_3 \circ C_3 \circ \texttt {Max} \circ C_1 ) (z_x) ) ), \end{aligned}$$

7 $$\begin{aligned} z_x&= \texttt {Reshap}( z_{1}; \ldots ; z_{K} ) , \end{aligned}$$

where $c_{t} (x) \in [0,1]$ is the output of a softmax function $\texttt {Sm}_t(x)$ as a confidence score for the class of *x* to be *t*, Mlp a multi-layer perceptron with two hidden layers of 256 neurons each, Avg a global average pooling, and Max a max pooling. Reshap stitches patch-level features $z_i$ from Eq. (4) into larger feature maps, $z_x \in {{\,\mathrm{\mathbbm {R}}\,}}^{1536 \times 44 \times 22}$, so that $z_i$ is moved back to the position of its corresponding patch in *x*. The goal of reshaping the patch-level features is to provide the model with the whole-image view for learning the positional prior of the lesions.

#### Localization

The autoencoder as a localization branch consists of an encoder *E* and a decoder *D*. The decoder takes as input $z_x$ with its reduced channel size from the classification branch, and combines the features with the encoding of *x* from *E* to predict a Gaussian heatmap $\hat{\psi }^s_x \sim \mathscr {N}(\mu _x, \sigma _x^2)$ as

8 $$\begin{aligned} \hat{\psi }^s_{x} = D ( \texttt {Concat} (E (x); z_x)). \end{aligned}$$

We use feature aggregation between layers of different levels^53^ to improve lesion segmentation of variable sizes. We use a single variance for heatmaps predicted from Eq. (8) regardless of lesion size. Then, a predicted lesion segmentation $\hat{s}_x = \{ \hat{s}_{x,i} = \mathbbm {1} ( \hat{\psi }^s_{x,i} \ge th_s ) \}$, with dimensions $w^\prime = 750$ and $h^\prime = 375$, is obtained by thresholding pixel-wise intensity $\hat{\psi }^s_{x,i} \sim \hat{\psi }^s_x$ with a threshold, $th_s$, where $\mathbbm {1}(\cdot )$ is the indicator function. We found that using heatmaps with lesion size-dependent variances tends to degrade segmentation performance, as heatmap activation decreases rapidly for smaller lesions.

### Data augmentation and loss functions

#### LesionMix for data augmentation

**Figure 2:**
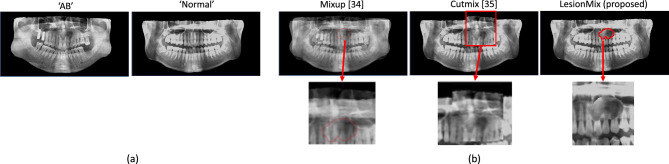
LesionMix, proposed data augmentation: (**a**) two samples of “AB” and “Normal” cases to generate a synthetic “AB” sample, and (**b**) comparisons of the result of LesionMix with that of Mixup^45^ by mixing the sources in the ratio 0.5:0.5 and Cutmix^46^. For each method, crops containing lesions are highlighted.

We propose a simple but effective way, which we call *LesionMix*, to augment training samples in response to class imbalance. In particular, we create new data samples for a minor class, e.g. “AB”, by pasting a cropped lesion from a minor class sample onto a normal sample, as shown in Fig. 2a. LesionMix aims to synthesize natural looking data samples in terms of texture and structure. In contrast, augmentation using Mixup^45^ may disrupt the tissue texture of lesions due to interpolation with normal tissue, as shown in Fig. 2b. Cutmix^46^ crops the lesions as rectangles rather than actual boundaries. Such cropping can lead to non-trivial misalignments in the anatomical structure of the jaws and provide incorrect signals to the model during training. Our experiments show that applying data augmentation with LesionMix when training DOLNet significantly improves performance.

#### Loss functions

Let $\mathscr {D}_{tr} = \{ (x, t_x, s_x) \}$ be a set of $N = \vert \mathscr {D}_{tr} \vert$ training samples. $t_x$ and $s_x$ are the target class and lesion segmentation maps of *x*, respectively. A loss function $\mathscr {L}$ for training $f_\theta (x)$ is given by

9 $$\begin{aligned} \mathscr {L} = \lambda _{cls} \mathscr {L}_{cls} + \lambda _{loc} \mathscr {L}_{loc}, \end{aligned}$$

where $\mathscr {L}_{cls}$ and $\mathscr {L}_{loc}$ are loss terms for the classification and the localization with weights of $\lambda _{cls}$ and $\lambda _{loc}$, respectively. $\mathscr {L}_{cls}$ is the cross entropy as follows:

10 $$\begin{aligned} \begin{aligned} \mathscr {L}_{cls}&= - \frac{1}{N} \sum _{x \in \mathscr {D} } \sum _{t=1}^{4} \mathbbm {1} ( t = t_x ) \log P_{t}(x), \end{aligned} \end{aligned}$$

where $P_{t}(x)$ is the probability that the class of *x* is *t*. Also, $\mathscr {L}_{loc}$ is given by

11 $$\begin{aligned} \mathscr {L}_{loc}&= \lambda _{h} \mathscr {L}_h + \lambda _{s} \mathscr {L}_s, \end{aligned}$$

12 $$\begin{aligned} \mathscr {L}_h&= \frac{1}{N} \sum _{x \in \mathscr {D} } \sum _{\hat{\psi }^s_{x,j} \in \hat{\psi }^s_x} \left( \psi ^s_{x,j} - \hat{\psi }^s_{x,j} \right) ^2, \end{aligned}$$

13 $$\begin{aligned} \mathscr {L}_s&= - \frac{1}{N} \sum _{x \in \mathscr {D} } \sum _{\hat{s}_{x,j} \in \hat{s}_x} (1 - p_t)^\gamma \log p_t, \end{aligned}$$

where $p_t = \hat{s}_{x,j}$ if $s_{x,j} = 1$ otherwise $p_t = 1 - \hat{s}_{x,j}$. $\mathscr {L}_h$ is the mean squared error of the heatmap prediction, and $\mathscr {L}_s$ is the focal loss^26^ to measure the prediction accuracy of the lesion segmentation. $\lambda _{h}$ and $\lambda _{s}$ are the weights of $\mathscr {L}_h$ and $\mathscr {L}_s$.

## Results and discussion

We conduct extensive experiments to answer the following questions:

- Is the diagnostic performance of DOLNet superior to that of previous work and even human clinicians? If so, how do the components of the proposed model contribute?
- Is the classification and localization of DOLNet consistent?
- What are the effects of the lesion size and location on the diagnoses of the proposed method and previous studies?

### Dataset

For this study, Jeonbuk National University Dental Hospital provided a dataset collected from 2000 to 2019 with the approval of the Institutional Review Board (approval number: 2019-05-057). The dataset contains 565 grayscale panoramic radiographs, 3000 $\times$ 1500 in size, of the upper and lower jaws of patients aged 15–81 years. We denote this dataset as $\mathscr {D}$, which consists of 75 AB, 97 OKC, 193 DC, and 200 normal cases. The abnormal cases were diagnosed based on histopathological findings. In general, AB and OKC are relatively rare compared to DC, and they are also difficult to distinguish from each other. Note that our model is generic enough that a new dataset with multiple lesions of different types can be used without changing the network structure or the loss functions.

**Figure 3:**
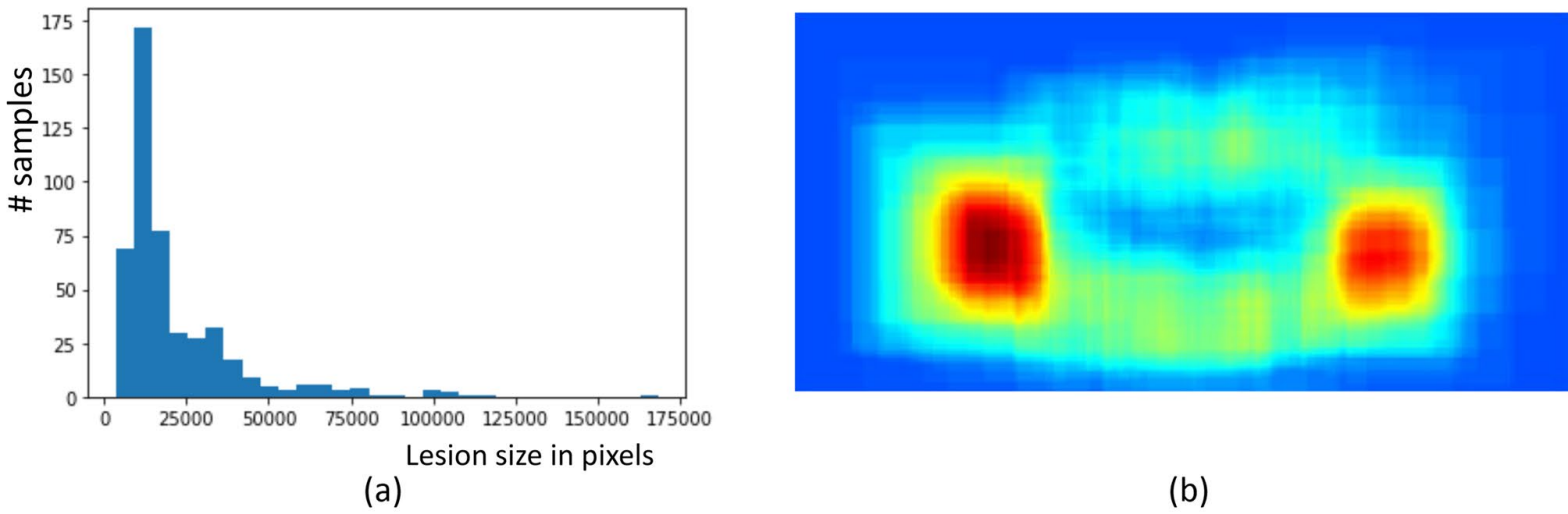
Characteristics of a dataset for this study: (**a**) the distribution of lesion size in the images, and (**b**) the spatial distribution of lesions across the images in terms of the normalized heat map corresponding to all lesion segmentations in our dataset (best viewed in color).

The normal samples were randomly selected and reviewed by two specialists with 6 and 15 years of experience. Fig. 3a,b show the distributions of lesion sizes and locations for $\mathscr {D}$, respectively. We preprocess the dataset by removing the background around the jaws and normalizing the images as shown in Fig. 1c. The mean and standard deviation of the color intensities of the images were set to zero and one, respectively.

Our dataset does not contain annotations for normal case images regarding lesion localization. Thus, the loss calculation during training is not affected by the localization prediction on normal case images. DOLNet produces a heat map prediction for lesion identification even for images diagnosed as normal case. In such a case, we ignore the heat map and only care about the lesion type.

### Setups

#### Training method

We train DOLNet in two steps. First, we fine-tune the backbone, i.e., DenseNet-121, which was pre-trained with ImageNet^54^. In particular, the backbone takes as input random crops of 375 $\times$ 375 from the training samples to predict corresponding lesion segmentations. Since naive random crops would lead to unbalanced sampling between lesional and non-lesional regions, we sampled crops that overlapped lesions three times more often than other regions. Given the input crops with the same loss function as Eq. (13), we then evaluated the prediction of Gaussian heatmaps with the distribution as Eq. (8).

Once the backbone is been fine-tuned, the second step is to perform end-to-end training of the model using the loss function in Eq. (9) with the parameters of the backbone fixed.

#### Hyperparameter

To fine-tune the backbone, we used an exponential learning rate schedule^55^ as $\mu _{t+1} = \gamma \cdot \mu _t$, where $\mu _{t}$ is the learning rate at the *t*-th epoch, with initial values of $\gamma = 0.98$ and $\mu _0 = 0.0001$.

When training the entire model, we set all of the coefficients in Eqs. (9) and  (11) to 1.0 after an extensive hyperparameter search. The parameters of the Gaussian distribution for a lesion heatmap of *x*, $\mu _x$ and $\sigma _x^2$, were set to the center of a bounding box surrounding a lesion and $\sigma _x^2 = 50$, respectively. The threshold, $th_s$ for the segmentation prediction, was set to 0.5. The split ratio of the datasets for training, validation, and test was 0.75:0.10:0.15. We also used fivefold cross validation to measure performance on the test set. We used the same learning rate scheduling strategy as for backbone fine-tuning, but with $\mu _0 = 0.0005$.

We implemented the model using PyTorch 1.7.1 and used six Nvidia RTX 3090 GPUs to train the model for 200 epochs. The size of a minibatch was 48 samples. We oversampled offline to balance the lesion categories, resulting in 200 ABs, 194 OKCs, 193 DCs, and 200 normal cases. During training, each minibatch retained the same number of original and synthesized samples as augmented by LesionMix at each online iteration. We also applied horizontal and vertical flips with the probabilities of 0.5 each, rotations of up to 5^∘^, and color jittering in [0, 1] for the brightness, contrast, and saturation, and in [0, 0.5] for the hue.

#### Performance evaluation methods

We use four metrics to evaluate the classification performance of DOLNet i.e., *precision*, *recall*, *accuracy*, and *F1 score*, and the intersection over union (IoU) for segmentation. We compared our approach with three recent works^6,11,19^. We carefully implemented the methods and trained them with $\mathscr {D}$. Since the recent method^19^ requires pre-training based on self-supervised learning with a large-scale dataset, we used 100 more normal samples in addition to $\mathscr {D}$ when training the model.

We also evaluate the performance of human clinicians, i.e., three oral and maxillofacial surgeons and two general practitioners, who were selected based on their areas of practice. They performed lesion classification on a randomly selected subset of $\mathscr {D}$ (denoted by $\mathscr {D}_{tiny}$) with 45 ABs, 59 OKCs, 120 DCs, and 120 normal samples. The test was performed individually for each physician, and the same data used for model validation was provided as JPEG images for evaluation. Intra- and inter-rater reliability was checked using sample data and confirmed to be greater than 95% before final evaluation.

### Quantitative evaluations

Tables 1 and 2 show the results of the quantitative evaluations by comparing the classification performance of DOLNet with that of the previous works on the dataset $\mathscr {D}$ and with that of human clinicians on $\mathscr {D}_{tiny}$. We also evaluate the effects of using the proposed augmentation technique, LesionMix, by comparing it with two existing techniques, Mixup^45^ with a ratio of 0.5:0.5 and Cutmix^46^. Overall, Table 1 shows that the proposed method achieves state-of-the-art results for all metrics by a considerable margin of 43.5% over the recent works^19^ and human clinicians by an average of 11.7%, respectively.

**Table 1:** (Upper part) Classification results of the state-of-the-art and variants of DOLNet on the dataset $\mathscr {D}$ and (lower part) human clinicians and DOLNet on $\mathscr {D}_{tiny}$.

Dataset	Method	Accuracy	Precision	Recall	F1 score
$\mathscr {D}$	Yang et al.^6^	0.499	0.467	0.490	0.478
	Kwon et al.^11^	0.503	0.447	0.436	0.441
	Hu et al.^19^	0.538	0.487	0.476	0.481
	Backbone-alt (ResNet-18)	0.581	0.493	0.509	0.501
	Backbone (DenseNet-121)	0.600	0.498	0.513	0.505
	Backbone + Patch-level Attention (PA)	0.649	0.648	0.656	0.652
	Backbone + PA + Global Attention (GA)	0.747	0.660	0.676	0.668
	Backbone + PA + GA + Mixup	0.749	0.663	0.676	0.666
	Backbone + PA + GA + Cutmix	0.752	0.677	0.677	0.675
	**Backbone + PA + GA + LesionMix (DOLNet)**	0.769	0.702	0.678	0.694
$\mathscr {D}_{tiny}$	Human clinicians	0.691	0.603	0.632	0.617
	**DOLNet**	0.734	0.667	0.700	0.683

**Table 2:** Classification performance on the individual lesions for ameloblastoma (AB), odontogenic keratocyst (OKC), and dentigerous cyst (DC).

	AB				OKC				DC				Normal			
	Acc.	Pre.	Rec.	F1	Acc.	Pre.	Rec.	F1	Acc.	Pre.	Rec.	F1	Acc.	Pre.	Rec.	F1
Yang et al.^6^	0.819	0.349	0.264	0.301	0.811	0.265	0.264	0.264	0.739	0.615	0.571	0.562	0.678	0.553	0.661	0.602
Kwon et al.^11^	0.835	0.345	0.267	0.301	0.747	0.265	0.268	0.267	0.736	0.628	0.560	0.592	0.687	0.549	0.650	0.595
Hu et al.^19^	0.841	0.377	0.307	0.338	0.756	0.305	0.331	0.317	0.763	0.673	0.596	0.632	0.716	0.588	0.670	0.626
Human clinicians	0.887	0.614	0.382	0.471	0.782	0.372	0.389	0.38	0.806	0.747	0.668	0.705	0.904	0.975	0.796	0.876
DOLNet	0.890	0.627	0.427	0.508	0.814	0.459	0.464	0.462	0.919	0.862	0.907	0.884	0.917	0.859	0.915	0.886

Human clinician results were obtained using $\mathscr {D}_{tiny}$.

As reported in Table 2, the previous methods suffer primarily in discriminating between AB and OKC cases due to the categorical rarity and visual similarity of the lesions. Our approach outperforms the other approaches and clinicians in classifying these lesions. In particular, the recall and precision of the two previous methods^6,11^ are only half that of ours, due to an increase in false positives and false negatives. Table 2 shows that the identification of AB and OKC cases remains a challenge even for human clinicians. For example, DOLNet improves the lesion classification performance by 19.2 % and 21.0 % in recall and F1 score for the OKC cases, respectively, compared to the human clinicians.

#### Classification and localization coherence

A meaningful diagnosis requires that the model is accurate for both classification and localization. Table 3 lists the results of classification, “Cls. only”, and localization, “Loc. only”, and their combination, “Cls. + Loc”. Among them, the classification corresponds to the results shown in Table 1. Table 3 shows that the proposed method has a strong coherence between the classification and localization predictions. The performance degradation in the joint task of classification and localization is only 2.4 % of the classification performance. We believe that this prediction consistency is mainly due to the sharing of the reshaped image features of the classification branch with the localization branch.

**Table 3:** Evaluation of the proposed method for three task settings in terms of accuracy: classification only, localization only, and combined classification and localization.

	AB	OKC	DC	Normal	Acc.
Cls. only	0.427 (32)	0.464 (45)	0.907 (175)	0.915 (183)	0.769
Loc. only	0.893 (67)	0.814 (79)	0.917 (177)	–	0.926
Cls. + Loc.	0.413 (31)	0.433 (42)	0.870 (168)	0.915 (183)	0.750

When calculating the accuracy, we consider the localization successful if the corresponding IOU is greater than 0.5. Values in brackets are the number of samples corresponding to each of the lesion types.

Notably, the performance of the independent localization task with our approach is superior to that of the classification and joint task. Such a high accuracy of the localization task is beneficial for effectively identifying suspected lesion regions in the early stages of disease. This result also implies that lesion classification is more challenging than localization. For example, if we relax the classification task by considering AB and OKC as the same class, the four metrics listed in Table 1 for the combined class are 0.871, 0.832, 0.721, and 0.773, respectively, improving the precision and recall scores by up to 81.3% and 68.9%, respectively, compared to the case of the four categories.

#### Ablation study

We perform an ablation study to examine the impact of several key components of DOLNet on classification performance. The last five rows of the section “$\mathscr {D}$” in Table 1 correspond to the variants of DOLNet, which are the backbone selection, the use of patch-level feature learning with local attention, and the use of global attention, and the full configuration of DOLNet, respectively.

First, the DenseNet-121 backbone (’Backbone’ in the table) with 8 million weights outperformed ResNet-18 (’Backbone-alt’) with 12 million weights. Second, our choice of network structure, patch-based analysis with local attention, resulted in a significant boost in all metrics. In particular, the largest performance gain of 57.7 % appears in the recall compared to the previous works. As a result, our method robustly identified the anomalous cases and thus improved the classification of the AB and OKC cases in the presence of categorical rarity. Moreover, the use of global attention improves the performance, especially in terms of accuracy and recall. These performance gains demonstrate that the proposed hierarchical attention effectively identifies abnormal cases and reduces false negatives. Finally, the proposed data augmentation LesionMix leads to an additional significant performance improvement, especially in precision (up to 6.4%). The augmented data samples produce fewer false negatives by better detecting lesions with challenging spatial conditions.

To summarize, the above observations demonstrate the advantages of the proposed method in various aspects of performance.

### Effects of lesion size and location on diagnosis

**Figure 4:**
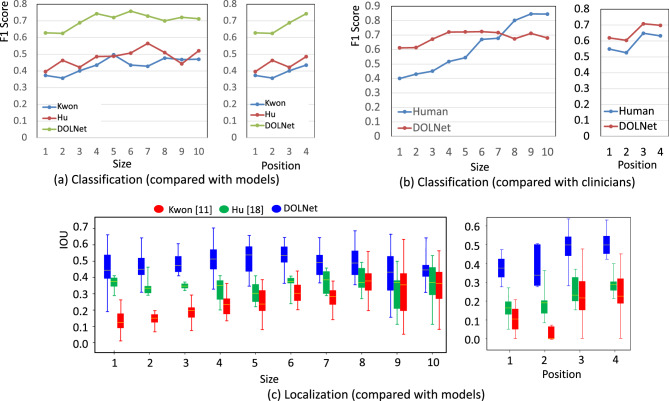
Effects of lesion size and location on classification by comparing the proposed method with (**a**) existing models, Kwon^11^ and Hu^19^ using the dataset $\mathscr {D}$ and (**b**) human clinicians using the dataset $\mathscr {D}_{tiny}$ and on localization by comparing with (**c**) the models. For the three plots with their horizontal axes labeled as *size*, the numbers correspond to identifiers of image groups (i.e., bins) according to the lesion size. Bins with larger ids contain images with larger lesions. Also, for the plots with the horizontal axes labeled as *position*, the numbers correspond to the four quadrants.

Figure 4 shows the effect of lesion size and location on the diagnostic performance of the models and human clinicians. First, we equally divided the test images into ten groups according to the number of pixels of the lesion areas in the images (the left plots in Fig. 4a–c). We also divided the entire region of a test image into four quadrants, with the image center as the origin. The quadrants were numbered from ${1}{\hbox {st}}$ to ${4}{\hbox {th}}$ in a counterclockwise direction, starting from the upper right quadrant (the right plots in Fig. 4a–c. We then measured the averaged F1 score and IOU of the test images in each of the bins and the quadrants of the entire test images for classification and localization performance, respectively.

The plots clearly show the advantage of the proposed method. While the classification performance of all the methods tends to deteriorate for smaller lesions, which is more significant in the previous works. Figure 4a,c shows that the previous works, especially Kwon et al.^11^, also suffer in localizing small lesions. While Hu et al.^19^ showed the consistent localization performance against variations in lesion size, its overall localization performance is inferior to DOLNet. Furthermore, the localization accuracy Kwon et al.^11^ decreases significantly in the ${1}{\hbox {st}}$ and ${2}{\hbox {nd}}$ quadrants corresponding to the upper jaw, where the incidence of lesions is relatively rare. In contrast, DOLNet shows robust and accurate localization against both size and position variations compared to the other methods.

We observed that the human clinicians were good at identifying large lesions, resulting in F1 scores greater than 0.8 for the last three bins containing large lesions, as shown in Fig. 4b. The slight decrease in performance in locating large lesions with our method is likely due to the lack of training samples. However, the clinicians had difficulty classifying small lesions. While the variations in localization accuracy according to lesion position are almost identical for our approach and the human clinicians, our method achieves higher localization accuracy than the clinicians in all regions.

### Effects of backbone nework structure

It is questionable whether the design choice regarding the backbone of DOLNet is optimal compared to the cases of using a backbone network beyond CNN or changing the receptive field size. To this end, we consider two candidates to replace the current DenseNet-based backbone of the proposed method. First, we take SwinTransformer^44^, a state-of-the-art model for various visual tasks, as the backbone. We use the tiny SwinTransformer, *Swin-T*, which is the smallest SwinTransformer model with 36M parameters, 3.6 times larger than the backbone of DOLNet. We keep the rest of the structure of the proposed method. As a result, the features fed into the decoder of the localization branch in Fig. 1b are given by Swin-T. As the second candidate, we consider mask R-CNN^24^, a popular model for detection and segmentation. We use ResNet-18^56^ as the backbone of the mask R-CNN model because its parameter size, which is 11M, is the most similar to our backbone among the ResNet variants. The receptive field size of ResNet-18 is 435 $\times$ 435, which is also similar to our backbone. Note that the mask R-CNN model takes the whole image as input, unlike our approach. As a result, the effective size of the receptive field with the mask R-CNN model for lesion prediction is reduced compared to our approach according to the number of patches for an input image. For lesion classification, we add a network branch consisting of two fully connected layers by taking $7\times 7$ convolutional features from the ResNet-18 backbone to the original mask R-CNN mode. Accordingly, we use the loss function to evaluate the classification error with the same weight as other loss terms corresponding to the segmentation. Swin-T and mask R-CNN were pretrained on ImageNet^54^ and MS COCO^57^, respectively. We train the models using the same procedure and hyperparameters as the proposed method.

Table 4 shows the effects of the backbone designs on lesion classification and localization. While SwinTransformer also learns hierarchical attention, the performance of the Swin-T based model is inferior to that of the proposed approach. The performance degradation is likely due to the fact that the dataset, $\mathscr {D}$, is insufficient to adequately train the Transformer-based backbone. This result suggests that, if carefully designed, the relatively small-capacity model could be beneficial for the problem addressed in this work, where scalable dataset acquisition is limited.

**Table 4:** Comparisons with two other models, a variant of the proposed model using the tiny SwinTransformer^44^, Swin-T, as the backbone and a mask R-CNN^24^.

	DOLNet with Swin-T^44^	Mask R-CNN^24^	DOLNet (proposed)
Accuracy	0.730	0.596	0.769
Precision	0.690	0.510	0.702
Recall	0.671	0.520	0.694
IOU	0.410	0.389	0.427

Rows 2–4 show the classification performance, while the last row corresponds to the segmentation for lesion localization.

Our model also outperforms the mask R-CNN-based model. This gain is mainly because, unlike the mask R-CNN, the proposed approach aggregates the patch-level analyses for an overall image-level prediction, even when the receptive field size of the backbones of the two models is similar. This evaluation demonstrates the validity of our design, which exploits the effectiveness of receptive field enlargement.

### Qualitative evaluation

**Figure 5:**
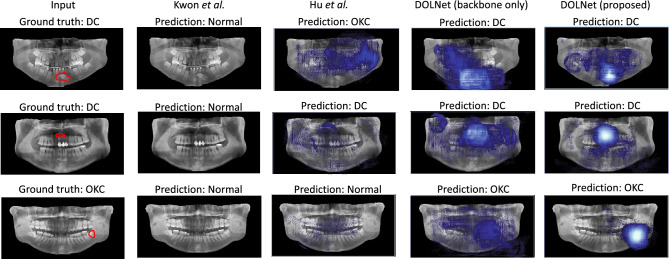
Qualitative results. Each of the rows contains a test image with its target class and lesion boundary annotated in red (${1}{{\hbox {st}}}$ column), and the predictions from four methods as blue heatmaps indicating lesions: Kwon et al.^11^ (${2}{{\hbox {nd}}}$), Hu et al.^19^ (${3}{\hbox {rd}}$), the backbone of DOLNet (${4}{{\hbox {th}}}$), and the full DOLNet (the last) (best viewed in color).

Figure 5 illustrates the results of qualitative evaluations on two works^11,19^ and our model using three challenging cases in the test dataset corresponding to each of the rows in the figure. In these cases, the lesions are small or in unusual locations. To highlight our patch-based analysis, we also evaluated a variant of our method that uses only the backbone and takes an entire image as input. We apply a simple decoder network to the last convolutional layers of the backbone to obtain the lesion segmentation.

Kwon et al.^11^ misclassified all examples as normal, so there are no bounding boxes. Hu et al.^19^ also resulted in incorrect predictions in the first and last cases or ambiguous localization in the second case. Our backbone-only model already outperformed the previous methods in the first two cases. However, all methods except the full DOLNet failed to localize the lesion in the last case, which contains a small lesion of the minor category. Furthermore, DOLNet localized the lesions of the first two examples in a more focused manner, supporting the effects of the proposed patch-based analysis.

## Conclusion

We proposed a novel method, DOLNet, to identify the three representative types of odontogenic lesions and their locations from a given panoramic radiograph. Mutually influencing hierarchical attention is the essential part of our approach, which extracts features at different scales, the whole image level and the patch level. We aim to build DOLNet so that the whole image representations allow the model to learn the global structure, while the model also learns the patch-level representations to discriminate between normal and abnormal tissue. We also introduced a simple yet effective data augmentation method, LesionMix, to synthesize the training samples of abnormal cases with realistic anatomical structures, thereby addressing the class imbalance problem.

Intensive experiments showed that DOLNet significantly outperformed recent methods in classification and localization. Furthermore, the classification performance of our approach was superior to that of experienced human clinicians. An ablation study showed that the hierarchical attention and LesionMix of our approach lead to a significant improvement in the diagnosis of challenging cases with lesions of small size or in unusual locations. The experimental results support the potential benefits of the proposed method for achieving augmented intelligence in the early diagnosis of odontogenic lesions.

Although the proposed method is promising, there is room for improvement. Similar lesion types such as AB and OKC are still difficult to identify from radiographs alone. A multimodal analysis including CT images may overcome this difficulty. Furthermore, the synthesis of training samples using a generative model to learn the anatomical structure and lesion tissues seems feasible.

## Data Availability

The datasets generated and/or analyzed in the current study are not publicly available due to an internal policy of Jeonbuk National University Hospital but are available from the corresponding authors upon reasonable request.
